# The efficacy and safety of Tocilizumab in adults with refractory systemic onset JIA

**DOI:** 10.1186/1546-0096-9-S1-P137

**Published:** 2011-09-14

**Authors:** G Deslypere, C Wouters, J Joly, R Westhovens

**Affiliations:** 1 Rheumatology, University Hospital KU Leuven, Belgium; 2 Pediatric Rheumatology, University Hospital KU Leuven, Belgium

## Objective

Systemic-onset JIA (so-JIA) is often persisting into adulthood and can be refractory to treatment including biologicals. We report on efficacy and safety of Tocilizumab in adults with persistent active so-JIA in a retrospective single center descriptive cohort study.

## Patients and methods

Disease activity scores (DAS-CRP), ESR and CRP were measured just before and at 1- 6- and 12 months of Tocilizumab treatment in all adults with refractory so-JIA at our University Hospital. Adverse events were recorded.

## Results

Eight patients (5 women, 3 men) with a median age of 27,5yr and median disease duration of 16 yr were treated. All previously failed Methotrexate, other classical DMARD’s, 7/8 also failed on one or more biologicals (mostly TNF blockers, Anakinra in 1). Median HAQ before starting Tocilizumab was 1.25 (0.75-2.13). Tocilizumab dose was 8mg/kg/month, except for 1 patient receiving 6mg/kg/2 weeks. The median DAS-CRP decreased from 5,10(1.6-5.3) (n=6) at start to 2,25(1.3-2.9) (n=6) after six months (p=0,046). At 12 months, median DAS-CRP was 1,9(1.5-6.1)(n=3). Median ESR/CRP decreased from 35(15-87)(n=6)/69,2(9.1-292.6)mg/l(n=8) at start to

2(1-7)/1,2(0.6-1.4)mg/l(n=7) at six months (p=0,028/0,018). At 1 year median ESR/CRP were 3,5(1-13)(n=4)/0,6(0.6-23.1)mg/l(n=5). A decrease/complete stop of steroids was observed in 6 patients. Atypical rash was seen in one patient, EBV-induced MAS occurred in 1 patient after 7 yrs of Tocilizumab.

## Conclusion

Our report on young adults with persistingly active so-JIA treated with Tocilizumab shows a fast, steep and persistent decrease in disease activity up to at least 1 year, demonstrating the efficacy of IL6-receptor antagonism also in patients with long-standing refractory so-JIA.

